# The iron(III) coordinating properties of citrate and α-hydroxycarboxylate containing siderophores

**DOI:** 10.1007/s10534-024-00607-z

**Published:** 2024-05-21

**Authors:** Robert C. Hider, André M. N. Silva, Agostino Cilibrizzi

**Affiliations:** 1 Institute of Pharmaceutical Science, King’s College London, London, SE1 9NH UK; 2 LAQV-REQUIMTE, Departamento de Quimica E Bioquimica, Faculdade de Ciencias, Universidade Do Porto, Rua Do Campo Alegre, S/N, 4169-007 Porto, Portugal

**Keywords:** Siderophores, α-hydroxycarboxylate, Iron, Citrate, Photo-induced decarboxylation, Algal iron uptake

## Abstract

The iron(III) binding properties of citrate and rhizoferrin, a citrate containing siderophore, are compared. Citrate forms many oligonuclear complexes, whereas rhizoferrin forms a single mononuclear complex. The α-hydroxycarboxylate functional group, which is present in both citrate, and rhizoferrin, has a high affinity and selectivity for iron(III) under most biological conditions. The nature of the toxic form of iron found in the blood of patients suffering from many haemoglobinopathies and haemochromatosis is identified as a mixture of iron(III)citrate complexes. The significance of the presence of this iron pool to patients suffering from systemic iron overload is discussed. The wide utilisation of the α-hydroxycarboxylate functional group in siderophore structures is described, as is their photo-induced decarboxylation leading to the release of iron(II) ions. The importance of this facile dissociation to algal iron uptake is discussed.

## Introduction

Citrate is an essential metabolite in all living systems, by way of its central role in the Krebs cycle. It is also a strong ligand for the binding of metals (Shweky et al. 1994; Matzapetakis et al. 2000a, b). Citrate has a particularly high affinity for tribasic cations (for instance iron(III), gallium(III), aluminium(III), chromium(III) and rhodium(III) (Carrano et al. 1996a, b) by virtue of the presence of the α-hydroxycarboxylate functional group. Only tribasic cations are capable of facilitating the dissociation of this hydroxyl group (Martin 1986), dibasic cations such as zinc(II) lacking this ability at neutral pH values. As iron(III) is the dominant tribasic metal cation in biological tissue, the presence of the α-hydroxycarboxylate functional group in molecules, for instance citrate, renders them with a high selectivity for iron(III) (Silva et al. 2009a, b). By virtue of this property citrate is utilised by many bacteria and plant species for iron acquisition (Harle et al. 1995; Pitch et al. 1991). Indeed, a number of bacteria produce siderophores which feature citrate in the iron coordinating centre (Carrano et al. 1996a; Drechsel and Winkelmann 2000; Hider and Kong 2010).

In view of this central role of citrate and citrate-containing siderophores in the absorption of iron from the environment and subsequent distribution of iron within organisms together with the role of citrate in the formation of a toxic iron pool in some disease states, we considered it appropriate to overview the underlying chemistry centred on the interaction of iron with the α-hydroxycarboxylate functional group. The chemistry of iron(III) binding to citrate is complicated (Pierre and Gautier-Luneau 2000), but is somewhat simplified when the citrate moiety is incorporated into siderophore structures (Carrano et al. 1996a, b; Hider and Kong 2010).

## Protonation of citric acid (1) and rhizoferrin (2)

Citric acid (**1**) possesses three carboxylate groups and one hydroxyl group; the carboxylic acid functions possessing readily dissociable protons (pKa_1_ = 3.13, pKa_2_ = 4.76 and pKa_3_ = 6.40, Martell and Smith 1977). The hydroxyl group however possesses a higher pKa value, namely 14.4 (Table 1, Silva et al. 2009a). This pKa value is lower than that of simple aliphatic alcohols, due to the electron-withdrawing inductive effect of the α-carboxylate group. The presence of the β-carboxylate groups in citric and malic acids explains the enhanced reduction of the pKa value when compared to lactic acid (Table 1).

**Table 1:**
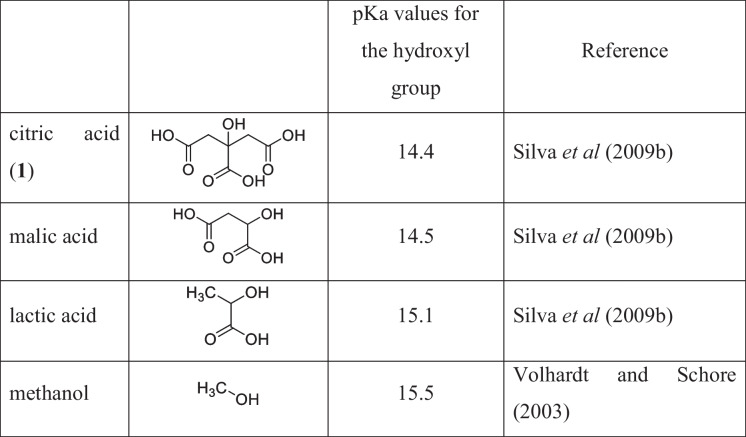
pKa values for the hydroxyl group in α-hydroxycarboxylic acids

**Figure:**
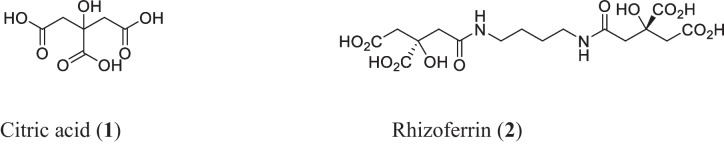

**Fig. 1:**
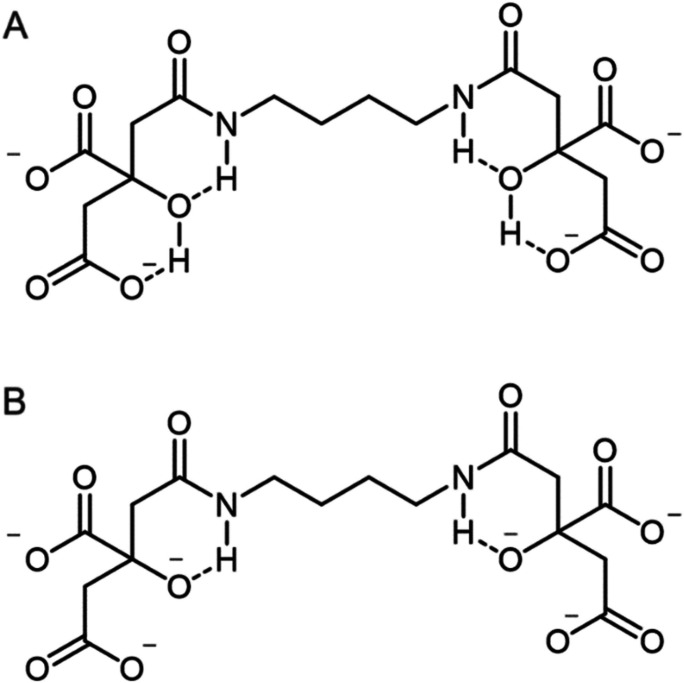
Schematic representation of intra-molecular hydrogen bonding likely to occur in rhizoferrin (**2**). A [rhizoferrin]^4−^ and B [rhizoferrin]^6−^

Interestingly Carrano and colleagues (1996a, b) reported somewhat lower pKa values for the two hydroxyl groups in rhizoferrin (**2**), when compared to the hydroxyl pKa value of citrate (Silva et al. 2009a). These two values, 11.3 and 10.05, were confirmed by Silva et al. (2009b). The increased acidity of the alcohol groups in this siderophore may be explained by the influence of intra-molecular hydrogen bonding. As noted by Sykes (1986), the effect of intra-molecular hydrogen bonding should not be disregarded in the energy stabilization of molecules. In rhizoferrin (**2**), two sets of intra-molecular hydrogen bonds are possible (Fig. 1). The hydroxyl groups may form hydrogen bonds with both the deprotonated carboxylate in the citric acid moiety and the amide hydrogen atom (Fig. 1A). This latter bond may also form with the hydroxyl anion formed upon the alcohol deprotonation (Fig. 1B). In cases where intra-molecular hydrogen bonding occurs in both the undissociated acid and the conjugate anion, the dissociation of the hydroxyl function will be facilitated (Sykes 1986). Such intra-molecular hydrogen bonding in rhizoferrin would stabilize the hydroxy anion, explaining the greater decrease in the pKa value. No such enhancement is possible with citric acid as all the carboxylate functions will be fully deprotonated at neutral to alkaline pH values.

Intramolecular hydrogen bonding almost certainly occurs in other α-hydroxycarboxylate-containing siderophores, which will lead to reduced competition between protons and iron(III) for binding to the α-hydroxyl-carboxylate function, thus enhancing the affinity for iron(III) under typical conditions found in the environment, ie freshwater, marine water, and physiological fluids.

## Chelation of iron(III) by citric acid (1) and rhizoferrin (2)

**Fig. 2:**
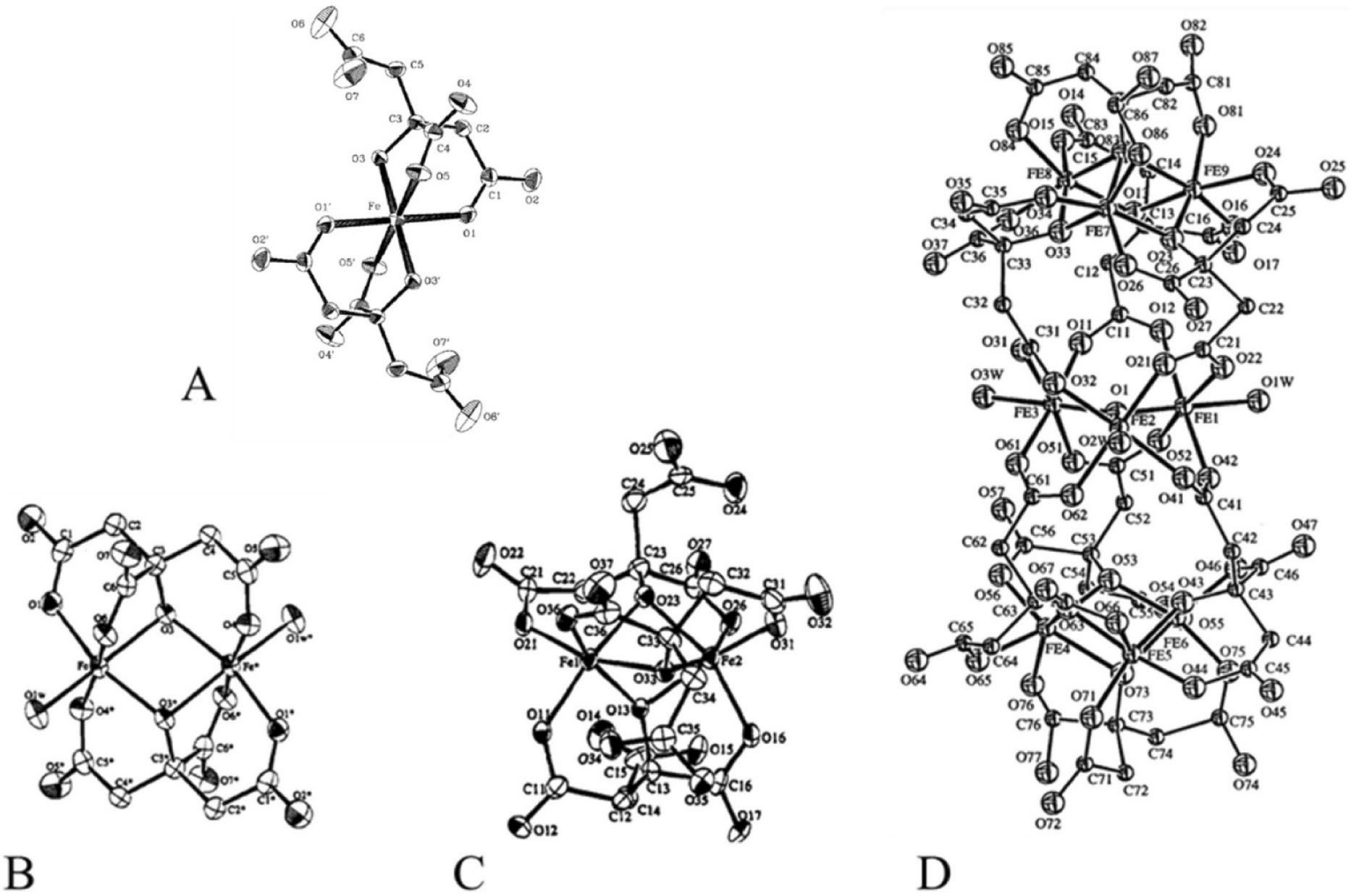
Xray crystallographic structures of iron(III) citrate complexes A, [Fe^III^(Cit)_2_]^5−^ from Matzapetakis et al. (1998); B, [Fe_2_^III^(Cit)_2_(H_2_O)_2_]^2−^ from Shweky et al. (1994); C, [Fe_2_^III^(HCit)_3_]^3−^ from Shweky et al. (1994); D, [Fe_9_^III^O(Cit)_8_(H_2_O)_3_]^7−^ from Bino et al. (1998). The ORTEP diagrams show the probability surface with thermal ellipsoids, reported at values of 50% for A, B, and 35% for C

**Fig. 3:**
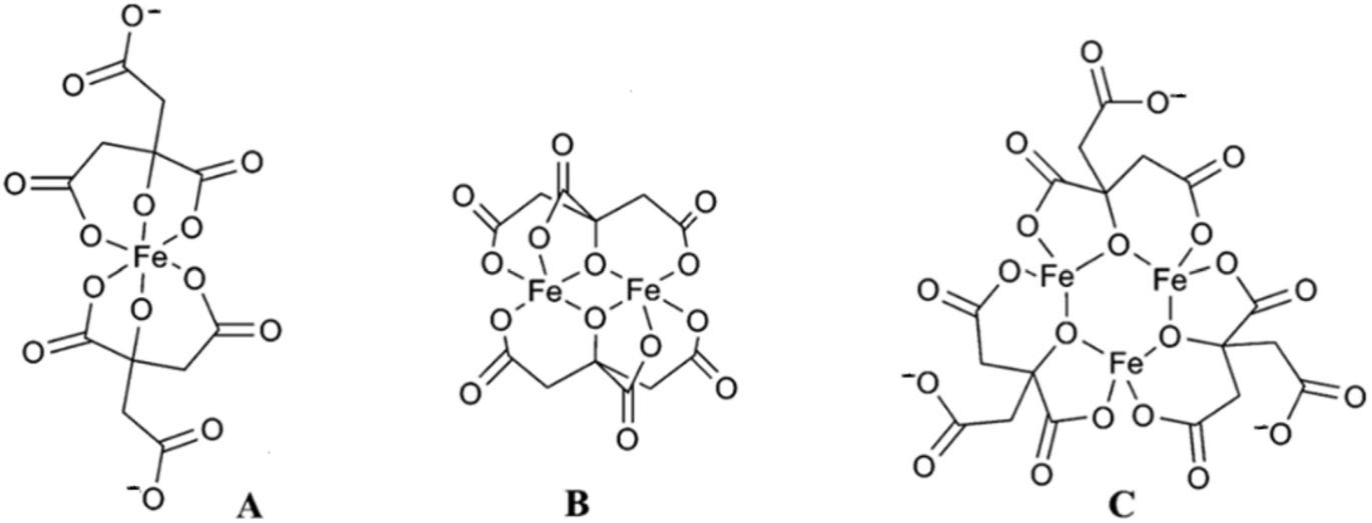
Schematic representation of the structure of the ferric citrate complexes existing in aqueous solution. **A** [Fe(Cit)_2_]^5−^, ML_2_; **B** [Fe_2_(Cit)_2_]^2−^, M_2_L_2_; **C** [Fe_3_(Cit)_3_].^3−^, M_3_L_3_

Iron(III) citrate chemistry is complicated by the ready formation of a range of oligomers. Xray crystallography data indicates that the alcohol function of citrate is directly involved in iron(III) coordination and that deprotonation of this functional group occurs on complex formation. A range of such complexes (Fig. 2) includes [Fe^III^(Cit)_2_]^5−^, [Fe_2_^III^(Cit)_2_(H_2_O)_2_]^2−^, [Fe_2_^III^(HCit)_3_]^3−^ and [Fe_9_^III^O(Cit)_8_(H_2_O)_3_]^7−^. With the mononuclear species (Fig. 2A), iron(III) is octahedrally coordinated by six oxygen atoms provided by the two coordinating citrate molecules. Each citrate provides 2 carboxylate and 1 alkoxy group. With the dinuclear species (Fig. 2B) both iron(III) ions are octahedrally coordinated by six oxygen atoms provided by two coordinating citrate molecules. The two alkoxide oxygens are shared by both iron(III) ions. The six carboxylate groups coordinate the two iron(III) centres together with two water molecules. A second binuclear complex coordinated by three citrate anions has also been crystalized (Fig. 2C). In this structure, the two iron(III) are coordinated by the α-hydroxycarboxylate moieties of each citrate, with their alkoxide functions bridging the metal ions and each citrate further contributing to coordination with another carboxylate group. Similarly to the mononuclear complex, one carboxylate in each citrate molecule does not participate in iron binding. The more complicated nonairon(III) citrate complex (Fig. 2D) essentially consists of three tri iron(III)-containing planes, the iron(III) ions in the top and bottom planes are octahedrally coordinated by 2 alkoxy oxygens and 4 carboxylate oxygens. Each of the iron(III) ions in the central plane are coordinated by 2 alkoxy oxygens, 3 carboxylate groups and 1 water molecule. Additional closely related oligomeric structures have also been reported (Pierre and Gautier-Luneau 2000). The aqueous speciation of iron(III) citrate has been investigated by mass spectrometry and EPR spectroscopy (Silva et al. 2009a). These studies have been interpreted to indicate that at high iron(III): citrate molar ratios (1:10 – 1:2) the speciation is dominated by oligomeric complexes such as the dinuclear and trinuclear species (Fig. 3B and C). The mononuclear dicitrate species (Fig. 3A) tends to dominate at low iron(III): citrate molar ratios (1:50 – 1:200). No crystal structure is available for the proposed trinuclear species (Fig. 3C) but its structure has been reported to resemble the planar regions contained in the nonanuclear complex (Fig. 2C) (Gautier-Luneau et al. 2005).

In contrast to citrate, rhizoferrin (**2**) only forms a single complex with iron(III) (**3**), which is mononuclear (Eq. 1) (Carrano et al. 1996a). No evidence for oligonuclear species was observed under a wide variety of conditions. While citrate can form octahedral 2:1 complexes with iron(III) using 2 carboxylate and the hydroxyl oxygen donor atoms, an additional carboxylate donor remains free and this can lead to the formation of oligonuclear species. With rhizoferrin, the equivalent carboxyl function forms an amide bond with diaminobutane and hence will not coordinate iron(III) ions under neutral aqueous conditions. The affinity constant of rhizoferrin for iron(III) is similar to that of hydroxamate-containing siderophores (Carrano et al. 1996a). Thus, in addition to the catechol and hydroxamate functions, the α-hydroxycarboxylic acid functional group has been selected by many microorganisms as a bidentate moiety suitable for siderophore construction. The specificity and mechanism of rhizoferrin-mediated metal ion uptake by both rhizoferrin-producing fungi and bacteria has been reported by Carrano et al. 1996b.

1 $${{\mathrm{Fe}}}^{3+}+{{\mathrm{Rhizoferrin}}}^{4-}\rightleftharpoons {\left[{{\mathrm{Fe}}}^{{\mathrm{III}}}\bullet {\mathrm{Rhizoferrin}}\right]}^{3-}+{2{\mathrm{H}}}^{+}$$

**Figure:**
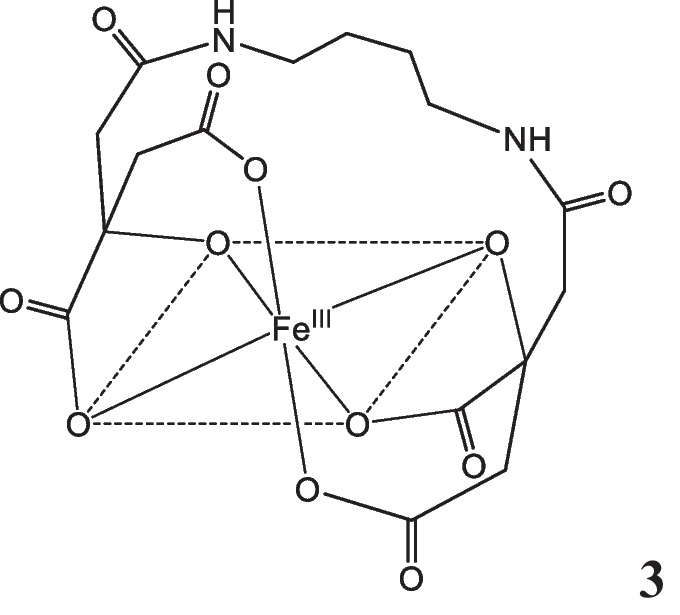

In order to indicate the importance of iron(III) coordination by the α-hydroxycarboxylate functional group in biological systems we describe two subjects in more detail, namely the role of nontransferrin-bound iron in a range of hereditary diseases and the biological properties of α-hydroxycarboxylate-containing siderophores.

### Nontransferrin-bound iron (iron(III)citrate) in patients suffering from systemic iron overload

Haemochromatosis is an inherited disease that is associated with systemic iron overload. Thalassaemia and sickle cell anaemia are also inherited diseases which frequently require regular blood transfusion as part of clinical treatment. Regular blood transfusion leads to systemic iron overload. Although iron is essential for life, it is toxic in excess. Systemic iron overload leads to saturation of circulating transferrin with iron, which in turn leads to the generation of a pool of nontransferrin-bound iron (NTBI) in the blood supply (Hershko 1975; Graham et al. 1979). This renders patients susceptible to infection and also causes an abnormal distribution of iron to various internal organs, in particular the heart and endocrine tissue. This maldistribution has severe consequences. Management of such patients requires that NTBI should be reduced to a minimum at all times, day and night.

Blood contains citrate at a level between 100 and 150 µM and it has been demonstrated to be a major ligand of nontransferrin-bound iron (Grootveld et al. 1989; Evans et al. 2008). The concentration of NTBI reaches levels up to 10 µM (Porter et al. 1996), thus indicating that the molar iron:citrate ratios fall close to 1:10. A mixture of iron(III)citrate complexes (Fig. 3) form under these conditions. Iron chelators employed clinically to remove iron from systemically iron-overloaded patients, for instance, desferrioxamine, deferiprone and desferasirox are all capable of removing iron from citrate complexes whether free in solution or bound to albumin (Evans et al. 2008). The removal of this toxic iron pool prevents the inappropriate build up of iron in the heart and endocrine tissue and is a key component in the treatment of patients suffering from systemic iron overload.

### α-Hydroxycarboxylate-containing siderophores

**Fig. 4:**
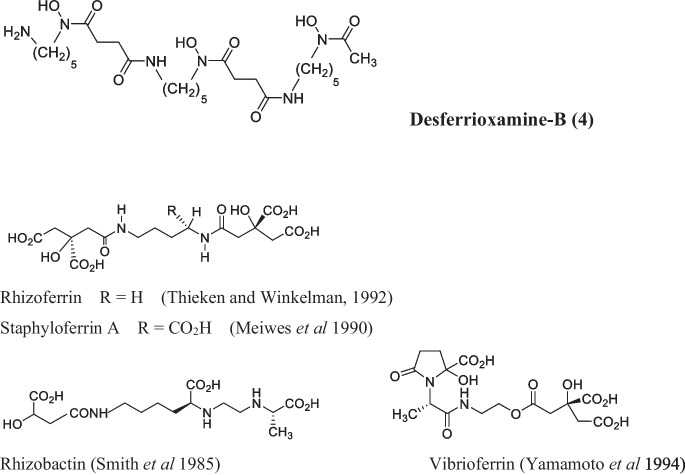
α-Hydroxycarboxylate-containing siderophores with terminal citrate or hydroxy succinate moieties

**Fig. 5:**
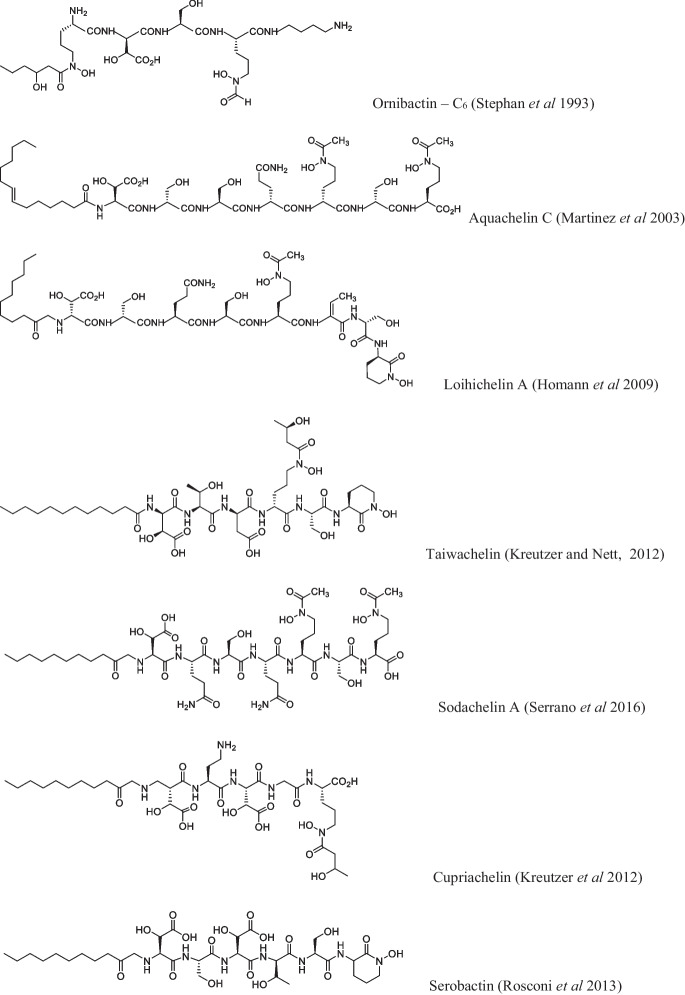
α-Hydroxycarboxylate/hydroxamate containing siderophores

There are a relatively large number of α-hydroxycarboxylate-containing siderophores (Figs. 4 and 5, Table 2), many also utilising the hydroxamate ligand to coordinate iron (Fig. 5). In addition to citrate, hydroxysuccinate (Fig. 4), 2-hydroxy-5-oxoproline (Fig. 4) and β-hydroxyaspartate (Fig. 5) provide α-hydroxycarboxylate functional groups in siderophore structures. Not all the analogues of known structure are listed in Figs. 4 and 5 and Table 2; there are for instance, 6 analogues of both loihichelin and sodachelin; 4 analogues of aquachelin (Martinez and Butler 2007) and 3 analogues each of ornibactin, cupriachelin, serobactin, nannochelin, ochrobactin and snyechobactin (Butler 2023; Hider and Kong 2010).

**Table 2:**
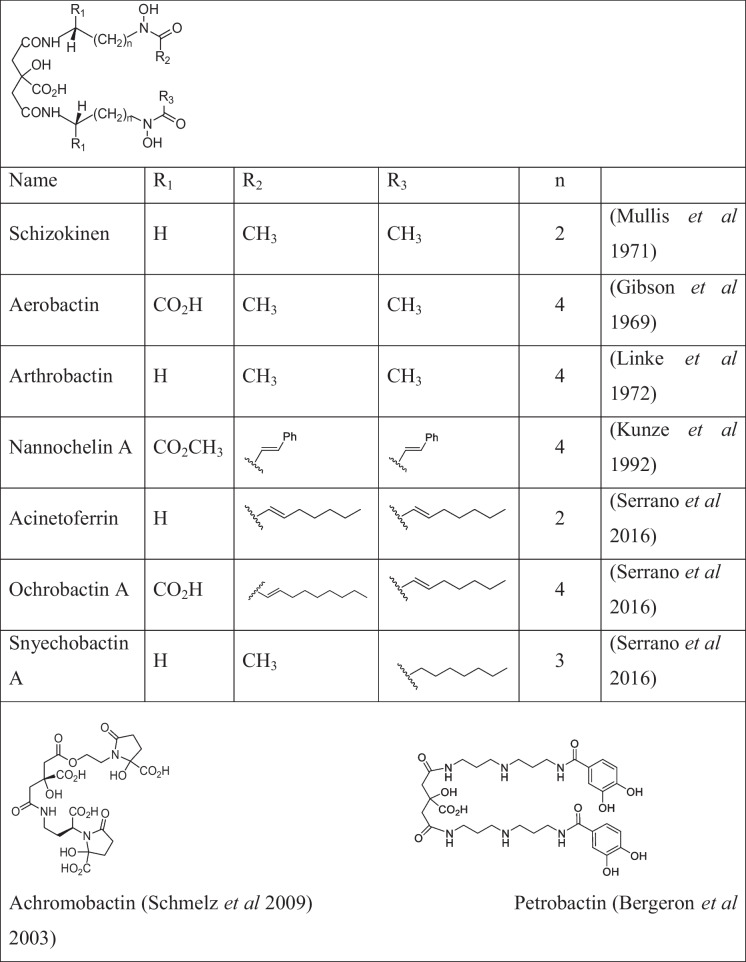
Siderophores containing a centrally placed citrate moiety

All siderophores listed in Figs. 4 and 5 and Table 2 are believed to act as hexadentate ligands for iron(III), but there has been relatively little work devoted to the structure of the corresponding complexes or indeed their coordination chemistry. Most of these studies have been undertaken by the Carrano group (Carrano et al. 1996a; Zhang et al. 2009). The affinity of the α-hydroxycarboxylate functional group for iron(III) is comparable to that of the hydroxamate group, judging by the pM values presented in Table 3, namely rhizoferrin (**2**), aquachelin C (Fig. 5), aerobactin, schizokinen (Table 2), and desferrioxamine B (**4**) (Carrano et al. 1996a; Chuljerm et al. 2019). The inclusion of the hydroxamate function in siderophore structures (Fig. 5) ensures that the net charge of the free siderophore is close to neutral and this probably accounts for the predominance of mixed hydroxamate and α-hydroxycarboxylate-containing siderophores, particularly in marine environments where siderophore lipophilicity appears to be critical for their ability to efficiently scavenge and donate iron to the bacteria (Martinez et al. 2000; Martinez and Butler 2007).

**Table 3:** Iron(III) – siderophore formation constants

	log K_ml_*	pM**	Reference
Aquachelin C	31.3	26.1^a^	Zhang et al. 2009
Rhizoferrin	25.3	19.7	Carrano et al. 1996a, b
Aerobactin	27.6	23.3	Harris et al. 1979
Schizokinen	36.2	26.8	Chuljerm et al. 2019
Desferrioxamine B	30.6	26.5	Anderegg et al. 1963

^*^K_ml_ = affinity constant of siderophore for Fe(III)

^**^pM = -log[Fe^3+^] where [Ligand]_Total_ = 10^–5^ M, [Fe]_Total_ = 10^–6^ M and pH = 7.4

^a^pM value for Aquachelin C has been estimated assuming a p*K*a value for the hydroxyl group in the α-hydroxycarboxylate moiety similar to that found in rhizoferrin

It is highly significant that almost 50% of marine siderophores are amphiphilic, a far higher percentage than that found with terrestrial siderophores (Butler 2023). The amphiphilic nature results from the incorporation of a range of fatty acids in the siderophore structure (Fig. 5 and Table 2). This amphilic structure is associated with the ability of the siderophore to aggregate into micelles, many of the siderophores possessing low critical micelle concentrations. Micelle formation probably limits diffusion of siderophores away from the cell, a particular problem with marine and freshwater bacteria (Volker and Wolf-Gladrow 1999) Micelle formation also offers protection from proteolytic cleavage (Butler 2023).

Some bacteria produce siderophores with a large range of partition coefficients. A gradient of siderophores with differing hydrophobicities could extend from the basic lipid membrane, through the polysaccharide matrix of outer membranes to the external bulk medium. The more hydrophobic siderophores would be predicted to associate tightly to the membrane, whereas the more hydrophilic siderophores could be secreted by the bacteria to the outer surfaces of the organism. Transport of iron could then be accomplished using a “bucket-brigade” approach (Martinez et al. 2003), with the more hydrophobic siderophores finally facilitating trans-membrane movement of iron utilising a “flip-flop” mechanism.

## Photoreactivity of iron(III) siderophores

**Fig. 6:**
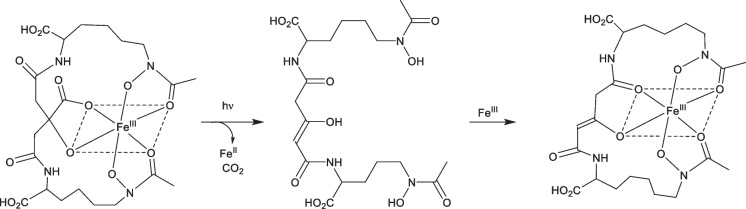
Photoreactivity of the aerobactin iron(III) complex. Adopted from Küpper et al. 2006)

The presence of α-hydroxycarboxylate groups in many marine bacterial siderophores renders their iron(III) complexes photoactive, as first reported by Butler and coworkers (Barbeau et al. 2001, 2002; Martin et al. 2006). The photoactivity of iron(III) aerobactin has been demonstrated to involve the decarboxylation of aerobactin and the simultaneous reduction and release of iron(II) from the complex (Fig. 6) (Küpper et al. 2006). Surprisingly the photo product, where the citrate fragment of the molecule is converted to the enol form of 3-ketoglutarate, is capable of coordinating iron(III) with high affinity, the resulting iron(III) complex being able to supply iron to bacteria in a similar fashion to that of the parent siderophore. A similar phenomenon occurs with petrobactin (Table 2) (Butler et al. 2021) and the ochrobactins (Table 2) (Martin et al. 2006). There are over 30 siderophore classes that possess photosensitive iron(III) complexes (Butler et al. 2021) and these can be subdivided into three main groups: (i) those possessing a central citrate moiety for example aerobactin (for others see Table 2); (ii) those with terminal citrate moieties such as rhizoferrin (for others see Fig. 4); and (iii) those with β-hydroxyaspartate moieties such as the aquachelin (for others see Fig. 5). The siderophores that lead to the formation of a photoproduct that binds iron(III) as well, or better than, the parent siderophore fall into the first class. Some siderophores yield a photoproduct that binds iron(III), but more weakly than the parent siderophores, for instance the aquachelins and some siderophores yield products that fail to bind iron(III) tightly. This latter group contains siderophores with the α-hydroxycarboxylate moiety in a terminal position (Fig. 4) and many of the β-hydroxyaspartate containing siderophores (Fig. 5), where photolysis generally leads to the cleavage of the peptide backbone (Butler et al. 2021).

The photoactivity of the iron(III) complexes of marine bacterial siderophores is obviously highly relevant to bacterial bioavailability of iron in marine and fresh water environments. Whether or not the differences in the iron binding properties of the photoproducts has any bearing on the mode and efficiency of bacterial iron uptake has yet to be established, but most certainly it is relevant to bacterial-algal mutualism in such environments. The influence of marine siderophores on the photochemical cycling of iron in oceans was first postulated by Butler and colleagues (Barbeau et al. 2001) and was subsequently followed up by Carrano and colleagues (Amin et al. 2009; Butler et al. 2021). Bloom-forming microalgae (phytoplankton), including dinoflagellates and coccolithophores, require close association with specific bacterial species, many of which produce α-hydroxycarboxylate-containing siderophores (Amin et al. 2009). The labile iron produced by the photolysis of iron(III) siderophore complexes becomes available to phytoplankton, which need iron in large amounts to support the photosynthetic fixation of carbon. This fixed carbon fuels the growth and reproduction of both phytoplankton and their bacterial associates. An analogous situation of mutualism involving the iron uptake by marine brown algae (Seaweed and Kelps) and bacteria was recently reported by the Carrano group (Cruz-Lόpez and Carrano 2023).

Although the precise mechanism of this tight interaction has yet to be elucidated, it is now clear that the sunlight-induced release of iron(II) facilitates the supply of iron to a wide range of algae and plays an essential role in the productivity of the oceans.

## Conclusions

Citrate itself can facilitate the uptake of iron in some organisms, it possessing a high affinity for iron(III) by virtue of its α-hydroxycarboxylate function. Citrate can also, under certain pathological conditions, bind freely available iron and adversely influence its normal distribution in a range of inherited diseases. Clinical treatment of β-thalassaemic patients has been designed to remove this toxic iron pool.

Whereas it has been long established that both the hydroxamate and catechol entities selectively bind iron(III) under most biological conditions (Raymond et al. 1984), the situation with the α-hydroxycarboxylate functional group has been less well established. However, in a similar fashion to hydroxamates and catecholates, α-hydroxycarboxylates form a very stable five-membered planar ring with iron(III). For many years the recognised two major classes of siderophore were hydroxamate- and catechol-based, even though several α-hydroxycarboxylate siderophores had been structurally characterised during the 1969 to 1972 period (Gibson and Magrath 1969, Mullis et al. 1971, Linke et al. 1972). More recently many more α-hydroxycarboxylate siderophores have been characterised, many being produced by marine bacteria (Butler 2023; De Serrano et al. 2016), clearly placing α-hydroxycarboxylate siderophores as a third major class.

The Butler and Carrano groups have demonstrated mutualism between marine bacteria that produce α-hydroxycarboxylate-containing siderophores and many different algae. Although the precise mechanism of this tight interaction has yet to be elucidated, it is now clear that the sunlight-induced release of iron(II) facilitates the supply of iron to a wide range of algae and plays an essential role in the productivity of the oceans.

## Data Availability

No datasets were generated or analysed during the current study.
